# Retraction Note: Integrated gene network analysis and text mining revealing PIK3R1 regulated by miR-127 in human bladder cancer

**DOI:** 10.1186/s40001-015-0119-3

**Published:** 2015-03-26

**Authors:** Yahong Xu, Shunwen Luo, Yang Liu, Jian Li, Yi Lu, Zhigang Jia, Qihua Zhao, Xiaoping Ma, Minghui Yang, Yue Zhao, Ping Chen, Yu Guo

**Affiliations:** Department of Urology, the 452nd Hospital of People’s Liberation Army, Chengdu, 610021 China; Cadre Aircrew Division, the 452nd Hospital of People’s Liberation Army, Chengdu, 610021 China; Nursing Department, the 452nd Hospital of People’s Liberation Army, Chengdu, 610021 China

## Retraction

The Publisher and Editor regretfully retract this article [1] because the peer-review process was inappropriately influenced and compromised. As a result, the scientific integrity of the article cannot be guaranteed. A systematic and detailed investigation suggests that a third party was involved in supplying fabricated details of potential peer reviewers for a large number of manuscripts submitted to different journals. In accordance with recommendations from COPE we have retracted all affected published articles, including this one. It was not possible to determine beyond doubt that the authors of this particular article were aware of any third party attempts to manipulate peer review of their manuscript.
